# Analysis of the Influencing Factors of the Efficient Degradation of Waste Polyurethane and Its Scheme Optimization

**DOI:** 10.3390/polym15102337

**Published:** 2023-05-17

**Authors:** Xiaohua Gu, Shangwen Zhu, Siwen Liu, Yan Liu

**Affiliations:** 1 State Key Laboratory for Modification of Chemical Fibers and Polymer Materials, College of Materials Science and Engineering, Donghua University, Shanghai 200051, China; glzsw2337899@163.com; 2 School of Energy and Building Environment, Guilin University of Aerospace Technology, Guilin 541004, China; 3 College of Innovative Material and Energy, Hubei University, Wuhan 430062, China; 202121113012770@stu.hubu.edu.cn

**Keywords:** waste polyurethane, duplex metal catalysts, alkali metal catalysts, co-catalysis, two-component alcoholysis agent

## Abstract

This work proposes an efficient catalytic recovery and utilization method for waste polyurethane foam. This method uses ethylene glycol (EG) and propylene glycol (PPG) as two-component alcohololytic agents for the alcoholysis of waste polyurethane foams. For the preparation of recycled polyethers, the conditions of different catalytic degradation systems were catalyzed by duplex metal catalysts (DMC) and alkali metal catalysts, and a synergy with both was also used. The experimental method was adopted with the blank control group and was set up for comparative analysis. The effect of the catalysts on the recycling of waste polyurethane foam was investigated. The catalytic degradation of DMC and the alkali metal catalysts alone, as well as the synergistic effect of the two catalysts, was explored. The findings revealed that the NaOH and DMC synergistic catalytic system was the best, and that the system activity was high under a two-component catalyst synergistic degradation. When the amount of NaOH added in the degradation system was 0.25%, the amount of DMC added was 0.04%, the reaction time was 2.5 h, and the reaction temperature was 160 °C, the waste polyurethane foam was completely alcoholized, and the prepared regenerated polyurethane foam had high compressive strength and good thermal stability. The efficient catalytic recycling method of waste polyurethane foam proposed in this paper has certain guiding and reference values for the practical production of solid-waste-recycled polyurethane.

## 1. Introduction

As the sixth largest polymer in the world, polyurethane is widely used in industrial production [1], equipment manufacturing [2], and social life [3], specifically in scenarios such as cold storage or cold chain transportation [4], building insulation [5,6], oil pipeline transportation [7], fabrics [8], artificial leather [9], coatings [10], asphalt modifier [11], biomedicine [12] and other industrial machinery [13]. In Agileintel Research [14], it was found that polyurethane materials are extremely promising, and that the market size of related products continues to rise with a current industry market value of USD 59.9 billion, which is expected to continue growing at a compound annual growth rate of 4.2%.

Polyurethane includes six main application forms: flexible foam [15], rigid foam [16], elastomers [17], coating agents [18], adhesives [19], and sealants [20]. According to the statistics of BCC’s research [21], the polyurethane flexible and rigid foam market value, in 2021, exceeds 50% of the total market value of polyurethane products. However, as the polyurethane foam industry is booming, its number of waste products and scrap rate are gradually increasing [22,23]. For example, the scrap rate in the production of refrigerators can reach 7% to 10%, and there is also a high scrap rate in the production of other products. At the same time, all kinds of waste polyurethane materials that have exceeded their service life, such as trimmings in the production process and aging products, are also increasing, with an annual average of approximately 20% to 30% of waste polyurethane products requiring disposal. Therefore, in the current environment of carbon neutrality [24] and carbon peaking [25], there is an urgent need for economic, green, and sustainable waste polyurethane treatment methods.

Currently, a large number of scholars and researchers have devoted themselves to studying the recycling methods of waste polyurethane, which has become a hot spot in the global industry. The main recycling methods for waste polyurethane include burial [26], incineration [27], physical pulverization [28], thermal treatment [29], chemical treatment [30] and biodegradation [31]. Since polyurethane-related waste is a thermosetting polymer material, traditional disposal methods, such as incineration [32], can cause great damage to the environment. These disposal methods generates large amounts of carbon dioxide and toxic gases that aggravate the global warming trend and increase the number of landfills [33], which are not easily degraded by waste polyurethane and can cause land and groundwater pollution. After mechanical crushing [34], the waste polyurethane can only be used as a filling material or as skeleton support, which have little value in terms of utilization. Waste polyurethane [35] contains fluorine, chlorine, and other plastics; as such, various toxic gases are generated during the catalytic heat treatment process. Certain scholars [36,37,38] have made great contributions to the practice of biodegradation for the green economy degradation of used polyurethane and have found that certain bacteria and fungi—especially those containing esterases, ureases, and papains—can break the ester bond in the chain. However, although Bacillus and Pseudomonas can biodegrade waste polyurethane, the degradation of polyurethane via bacteria is limited by environmental conditions and degradation time.

In comparison, chemical treatment methods are an ideal method for recycling. The known methods for the chemical degradation of polyurethane are hydrolysis [39], alcoholysis [30], aminolysis [40], glycolysis [41], acidolysis [42], and esterolysis [43]. Among them, alcoholysis, as one of the most widely used methods for the chemical recycling of polyurethane waste foam, uses small-molecule alcohols as alcohololytic agents to degrade polyurethane into polyether polyols in the temperature range of 150–300 °C. Although the cost of polyurethane alcoholysis recovery is inexpensive, green and economical, as well as its reaction environment being suitable for large-scale recovery, it has a relatively small recycling efficiency. Certain scholars [44,45,46] have found that catalysts can play a great role in the thermal degradation of polyurethane. However, there are relatively few studies on catalysts in the chemical degradation of waste polyurethane degradation, especially with respect to alcoholysis. Moreover, for traditional alkali metal catalysts, catalytic degradation efficiency is low; as such, it is important to research and develop new efficient catalysts for the degradation of waste polyurethane [47,48,49].

In this paper, the use of high-efficiency catalysts for the degradation of polyurethane is discussed. The bimetallic catalyst DMC was compared with the traditional catalyst for application and analysis. It has a rapid catalytic effect on the degradation system, higher catalytic activity than the alkaline catalyst, and has low requirements for the reaction environment, which is suitable for continuous production. This can solve the problem in which it is difficult to effectively treat waste polyurethane. Moreover, the new catalyst is green and environmentally friendly, and the recycling of waste polyurethane foam results in pollution and CO_2_ reduction, which is of great strategic significance to the sustainable development of human around the world.

## 2. Materials and Methods

### 2.1. Materials and Reagents

The materials and chemical reagents used in this experiment mainly include waste polyurethane rigid foam, waste plastic (Yantai, China); ethylene glycol, analytically pure (China Pharmaceutical Guangzhou Chemical Reagent Factory) (Guangzhou, China); propylene glycol, analytically pure (China Pharmaceutical Guangzhou Chemical Reagent Factory); DMC, homemade in the laboratory; NaOH, analytically pure (Beijing Kono Chemical Co., Ltd. (Beijing, China), Shandong Huasheng Chemical Co., Ltd. (Zhanhua, China); tin solution, 93% (China Pharmaceutical Guangzhou Chemical Reagent Factory); foaming agent CAS287-92-3, industrial grade (Shandong Huasheng Chemical Co., Ltd.); polyether 4110, analytically pure (Shandong Huasheng Chemical Co., Ltd.); and methylene diphenyl diisocyanate (MDI), industrial grade (Shandong Lianchuang Chemical Co., Ltd.).

### 2.2. Analytical Instruments and Methods

#### 2.2.1. Fourier-Transform Infrared Spectrometer (FTIR)

The chemical structure of the products after the degradation of waste polyurethane was analyzed using an FTIR. The analytical method was mainly used to prepare the samples via the press method, and the samples were tested in the wavelength range of 400–4000 cm^−1^.

#### 2.2.2. Scanning Electron Microscope (SEM)

To assess the waste polyurethane degradation in the foam after the recycled PU products were crushed into powder specimens, scanning electron microscopy was used for clear magnification to examine the structural integrity and uniformity of the region in order to observe the microstructure, the observation results were also recorded.

#### 2.2.3. Thermal Gravimetric Analyzer (TG)

The sample, after the degradation and regeneration of the waste polyurethane, was placed in a flowing argon atmosphere (140 mL·min^−1^) and heated to 500 °C at 10 °C·min^−1^, the weight change curve was also observed.

#### 2.2.4. Other Analytical Instruments

The other major experimental apparatuses used in this experiment included an analytical balance, Balance XPR106DUHQ/AC (METTLER TOLEDO Group, Zurich, Switzerland); a rotary viscometer, NDJ-5 (Brookfield Ametek (America) Co.) (Middleborough, MA, USA); a fully automatic true density analyzer, BELPYCNO L (Microtrac MRB (USA) Co.) (York, PA, USA); pull/pressure testers, F1505 (Mark-10 (America) Co.)(New York, NY, USA); and a disposable plastic cup, 350 mL (Depo Daily Chemicals (China) Co.) (Shanghai, China).

### 2.3. Analytical Instruments and Methods

**Figure 1:**
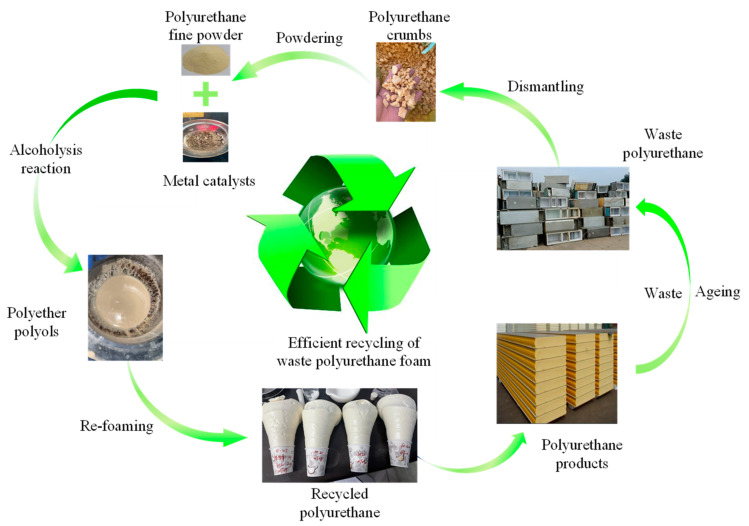
Efficient recycling of waste polyurethane foam.

The process for the efficient recycling of waste polyurethane foam is shown in Figure 1. The process is divided into two parts: the efficient catalytic degradation of waste polyurethane foam and the recycled polyurethane foam. The efficient catalytic degradation of waste polyurethane foam recycles and dismantles the waste that contains polyurethane foam, such as refrigerators, mattresses, and pipe insulation. The disassembled polyurethane foam is crushed and powdered, and then subjected to an alcoholysis reaction with polyol to produce polyether polyol. Recycled polyurethane foam and the recycled polyether polyol reacts with ethyl isocyanate in order to obtain recycled polyurethane foam.

#### 2.3.1. Analysis of the Mechanism of Waste Polyurethane Catalytic Degradation

The steps for the catalytic degradation of used polyurethane foam in this experiment were as follows: Firstly, the used polyurethane foam was crushed and processed into 15 mm particles. Then, the small-molecule alcohols EG and PPG were proportioned according to the ratio in Table 1, and their masses totaled 100 g. They were then added to the spherical glass reactor with different proportions of NaOH (an addition amount of 0.5%) and DMC (an addition amount of 0.04%) catalysts, as well as NaOH (an addition amount of 0.25%) and DMC (an addition amount of 0.04%) compound catalysts. They were then heated to 120–160 °C using an electric heating jacket, and the stirring bar was turned to 400 r/min for stirring while heating. Then, 100 g of crushed waste polyurethane foam was added, the temperature was raised to 160–220 °C, and then heated for 2.5 h in order to alcoholize it. After the complete alcoholysis reaction was cooled to room temperature, the alcoholysis product was filtered to obtain the regenerated polyether polyol. The regenerated polyether polyol was tested for hydroxyl value, viscosity, and infrared qualities. The reaction mechanism of this experiment was mainly that the waste polyurethane was synergized by the catalyst and alcoholysis agent to break the carbamate bonds in polyurethane and to replace them by small-molecule alcohol chains in order to obtain the recycled polyol. The recycling of the waste polyurethane foam was in line with the development concept of a circular economy and had the effect of energy saving and CO_2_ reduction, which is of great significance to the sustainable development of human, as well as for carbon peaking and carbon neutrality.

The traditional catalytic degradation mechanisms for polyurethanes are as follows:

R_1_-NHCOO-R_2_ + HO-R_3_-OH → R_1_-NHCOO-R_3_-OH + R_2_-OH (1)

Due to the large number of groups involved in the reaction during degradation, many side reactions will occur. The main side reaction is the cleavage of the urea group in the presence of alcohololytic agents to produce amines and polyols. The reaction process is as follows:

R_1_-NHCONH-R_2_ + HO-R_3_-OH → R_1_-NHCOO-R_3_-OH + R_2_-NH_2_ (2)

#### 2.3.2. Preparation of Recycled Polyurethane Foam

In this paper, in order to investigate the effect of regenerated polyether polyol—which was prepared by alcoholysis under different catalysts—on the performance of regenerated polyurethane foam, regenerated polyether polyol was prepared under the different conditions mentioned above; moreover, it was used as raw material to prepare the regenerated polyurethane foam. The preparation process was as follows:

In the first step, weigh 10 g of regenerated polyether polyol and 20 g of polyether polyol (4110) in a vacuum-drying oven. Dry and dehydrate at 80 °C for 1.5 h, then add the alcohol solution product and polyether polyol (4110) into a disposable plastic cup. Stir for 5 min with an electric mixer, and then place them in a vacuum-drying oven for a secondary dehydration for 1 h to obtain the mixture.

In the second step, weigh 50 g of diphenylmethane diisocyanate (MDI) in a vacuum-drying oven, and then dry and dehydrate at 80 °C for 1.5 h to process. Cool to room temperature, add the mixture in step one and stir at a constant temperature of 65 °C for 20 min for the pre-polymerization stage.

In the third step, the catalyst amine and tin, as well as the foaming agent (CAS287-92-3) and silicone oil were added to the disposable plastic cup. They were then stirred and mixed well, then left for 2 min. Next, they were mixed and stirred with the prepolymer in the above step after the mixture showed obvious delamination, and then stirred until a fine wire of 1 cm could be pulled out with a glass rod. After this, wait for the foam to grow continuously until the end of the foaming reaction. The prepared porous regenerated polyurethane foam was then recorded as regenerated polyurethane.

#### 2.3.3. Preparation and Catalytic Mechanism of DMC

Firstly, 100 mL of 0.8% ZnC1, 100 mL of 6% CaCl_2_, and 25 mL of tert-butanol were stirred and mixed to prepare mixed solution 1. Mixed solution 2 contained 25 mL 1.85% K_3_[Co(CN)_6_], and 25 mL 7% CaCl_2_ was stirred and mixed. Mixed solution 1 was added to a three-necked flask and placed on a thermostatic magnetic stirrer. The speed was set to 400 r/min and then it was stirred vigorously for 5 min. Next, this was then slowly added to mixed solution 2 dropwise in order to carry out the reaction. After the dropwise addition, 0.5 g of auxiliary complexing agent was added. Then, stirred rapidly for 10 min. When the reaction stopped the precipitate was separated by filtration through an extraction flask. The precipitate was first washed with 70% aqueous tert-butanol, then washed again twice with pure tert-butanol. Finally, the precipitate was placed in a vacuum-drying oven and dried at 50 °C to a constant weight in order to prepare the DMC. The mechanism of the reaction was as follows:

3ZnCl_2_ + 2K_3_[Co(CN)_6_] → Zn_3_[Co(CN)_6_]_2_ + 6KCl (3)

**Scheme 1:**
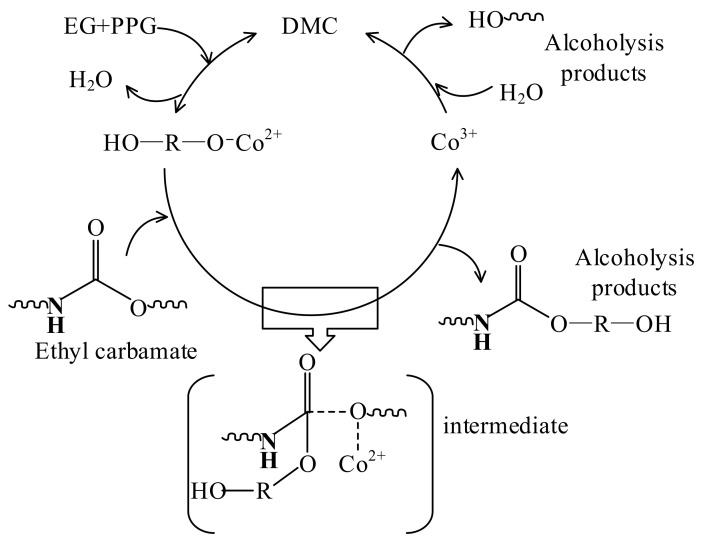
Catalytic mechanism of DMC.

DMC is a compound formed mainly from Zn and Co ions; furthermore, the high catalytic activity of DMC is mainly due to the presence of Co ions. As shown in Scheme 1, under high temperature conditions, regarding the process of the DMC-catalyzed degradation of the PU reaction, DMC produces an electron leap from Co^3+^ to Co^2+^ during the change in the reaction reduction. The surface electronic properties of the catalyst are then regulated and the catalytic activity improved. The surface active electron density center of the Co^3+^ shifts upwards, and the absorption capacity of the oxygen-containing groups is significantly enhanced, which greatly reduces the reaction potential, improves the activity of the catalyst, and promotes the carbamate bond breakage of polyurethane with less side reactions [50]. In contrast to conventional catalysts, Na^+^ and Co^3+^ ions will be present together in the composite catalyst of DMC and NaOH, where they are uniformly distributed, compensating for the uneven dispersion of a single catalyst ion in the solvent, thus resulting in a further enhancement of the catalytic activity of DMC, which is much greater than the catalytic activity of Na^+^ alone.

## 3. Results and Discussion

Based on the experimental steps described in the previous section, the effects of different alcohololyte formulations and catalyst formulations on the properties of waste polyurethane after degradation and regeneration were analyzed. In addition, the optimal formulations for the efficient catalytic degradation of regenerated polyurethane were investigated.

### 3.1. Analysis of Waste Polyurethane Foam Raw Materials

#### 3.1.1. Infrared Analysis

**Figure 2:**
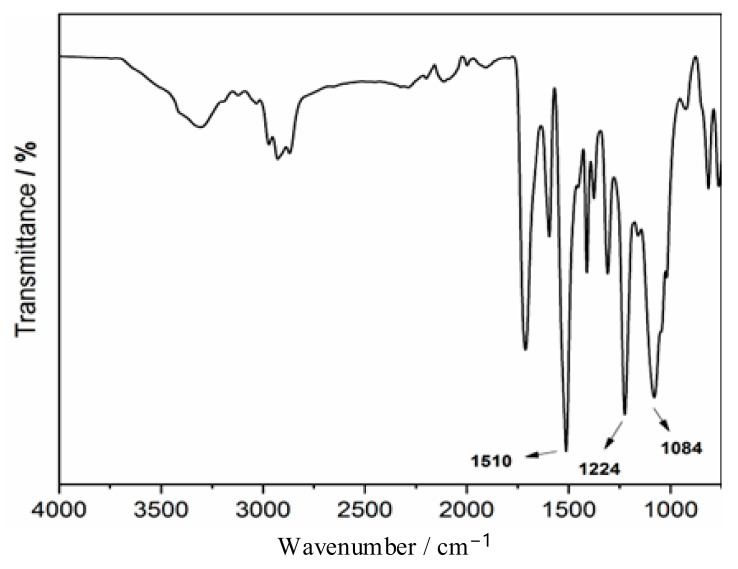
Fourier-transform infrared spectrum of waste polyurethane foam.

The raw materials of the waste polyurethane foam were identified and tested using FTIR spectroscopy. The IR spectra can be used to determine whether the isocyanate in the waste polyurethane foam is an aromatic or aliphatic isocyanate, and whether the polyol is a polyether polyol or a polyester polyol. This information is useful for analyzing and judging the degraded material after the alcoholysis of waste polyurethane. The infrared spectrum of waste polyurethane foam is shown in Figure 2.

As shown in Figure 2, there is a strong absorption band at 1510 cm^−1^ for the methylene (-CH_2_) absorption band between the two benzene rings. In addition, there is also a strong absorption band at 1224 cm^−1^ for the ester group (C=O) absorption band of aromatic polyurethane, as well as for the absorption band at 1084 cm^−1^ for the C-O-C group of the polyether-type polyurethane, such that it can be determined that the polyol used in the preparation of waste polyurethane foam is a polyether-type polyol and the isocyanate is diphenylmethane diisocyanate (MDI) or its prepolymer.

#### 3.1.2. Analysis of Differential Scanning Calorimetry

**Figure 3:**
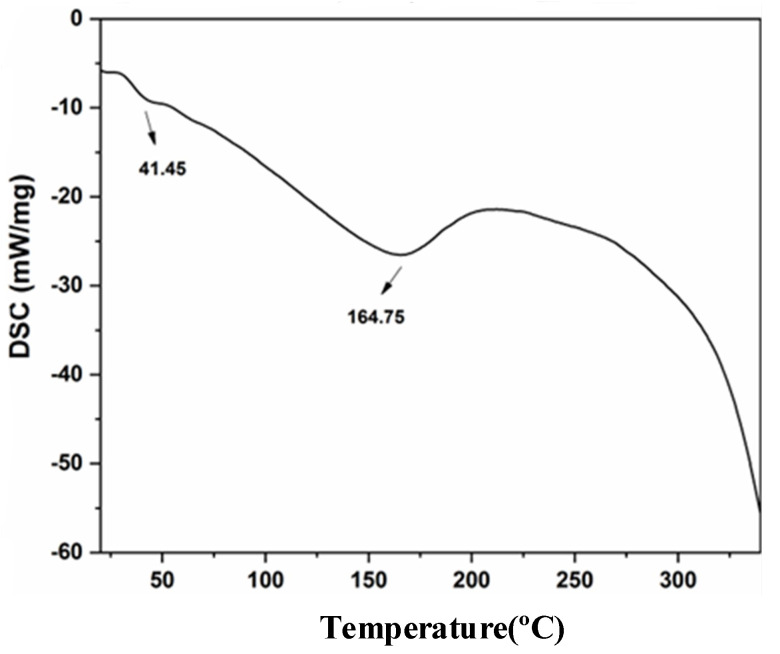
Thermal analysis diagram of waste polyurethane foam.

The differential scanning calorimeter was used for the thermal analysis of the waste polyurethane foam, and its test curve is shown in Figure 3. From Figure 3, it can be concluded that in the process of the differential thermal analysis of the raw materials of the waste polyurethane foam, the heat changes following the increase in temperature, which mainly exist in two places: the first place is approximately at 41.45 °C, which is the glass transition temperature of waste polyurethane foam; the other is approximately at 164.75 °C, which is the melting temperature of polyurethane foam. From the previous experimental conclusion, it is known that the alcoholysis temperature of polyurethane foam is fully degraded at 160 °C, which shows that the alcoholysis reaction of waste polyurethane foam is more easily carried out in the molten state.

### 3.2. Waste Polyurethane Degradation Material Situation

In this paper, the two components of ethylene glycol and propylene glycol were chosen as the alcohololytic agent, and the effect of different alcohololytic agents on the degraded material was studied. The reaction conditions were determined by pre-experiments as follows: the mass ratio of the waste polyurethane foam to the alcohololytic agent was 1:1, the addition of the alkaline catalyst NaOH was 0.5%, the mass fraction of the total mass of the reaction system was the same as below, the reaction temperature was 160 °C, and the reaction time was 3 h. The degradation materials are shown in Table 2.

As seen in Table 2, there are certain differences between the degradation materials of the two alcoholic solvents utilized in the alcoholic degradation of used polyurethane foam. The degradation materials of the propylene glycol and ethylene glycol are flowable liquid, and it is known from the theory that ethylene glycol and propylene glycol have high reactivity and degrade more completely. At the same time, waste polyurethane foam is used in a wide range of applications, mainly for thermal insulation. The synthesis scheme and ratio of polyurethane foam prepared for different applications are different. The ratio of degradation material and the ratio of foaming agents in the foaming process have a crucial influence on the physical and chemical properties (especially the strength) of the synthesized foam products. Choosing the right ratio of degradation material is of great significance for foam recycling and reuse. Therefore, propylene glycol and ethylene glycol are used as two-component synergistic degradation agents for the alcoholysis of used polyurethane foam in the degradation process of used polyurethane.

### 3.3. Effect of Two-Component Alcoholic Solvents on Degradation Materials and Analysis of Degradation Product Properties

**Figure 4:**
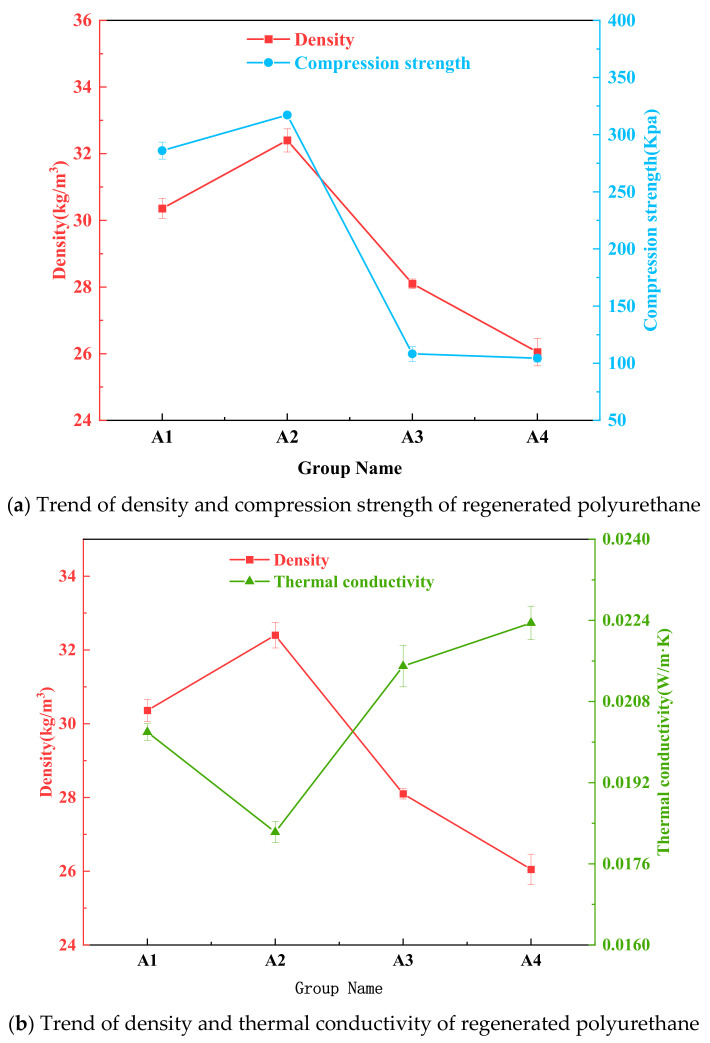
The relationship between the proportion of alcohol solvent and the performance of regenerated polyurethane.

As shown in Table 3, the viscosity of the degraded material and the performance of the recycled polyurethane foam after the degradation of the polyurethane with different ratios of alcoholic solvents was noted. According to the experimental data results, as shown in Figure 4, the viscosity of the degraded material will be affected by the composition of the alcoholic solvents. When the ratio of ethylene glycol and propylene glycol decreases, the viscosity of its degraded material first shows a trend of decreasing and then increasing. The reactant is over-degraded when the amount of propylene glycol is 1:1 with ethylene glycol, at which time the viscosity is the lowest at 1655.2 (Mpa·s, 25 °C) but also when the molecular weight is too low, which is not conducive to re-foaming. The degradation effect was also greatly reduced when the amount of ethylene glycol, another one of the alcoholic solvents used in the propylene glycol compounding, is too small. When the polyurethane macromolecular chain breakage is more uneven, resulting in a large number of unbroken long chains and in a higher viscosity of the degradation products, then this will also be detrimental to the foaming. Meanwhile, among the four groups listed in Table 3, the A1, A2, and A3 groups had a brownish color, while the A4 group had a golden color. The A1 and A2 groups had a fuller foam with a brownish color, and the A2 surface was flatter than what was seen in A1. The A3 and A4 groups saw difficulties in their foaming, and the individual successful ones shrank significantly after 24 h of standing. However, the foam was brittle, thus it was difficult to use in any practical applications.

The apparent density of the polyurethane foam, as an important performance reference index of polyurethane foam, directly affects the foam compression strength and thermal conductivity performance. Figure 4 shows the density and compression strength of the recycled polyurethane foam in relation to the compression strength and thermal conductivity. From Figure 4a, it can be seen that the density of the recycled polyurethane foam has a similar trend to the compression strength of the foam. In addition, the compression strength varies with the change in density. When the ratio of ethylene glycol to propylene glycol reaches 6:4 (A2), the compression strength and density reach the maximum, i.e., 317.2 KPa and 32.4 Kg/m^3^, respectively. According to Figure 4b, it can be seen that the density of the regenerated polyurethane foam shows a negative correlation with the trend of compression strength, and the thermal conductivity shows a trend of decreasing and then increasing as the ratio of ethylene glycol to propylene glycol in the alcoholic solvents decreases. When the ratio of ethylene glycol to propylene glycol reaches 6:4 (A2), the thermal conductivity of the regenerated polyurethane reaches the minimum, which is 0.01823 W·(m·K)^−1^. Therefore, the apparent density of the preferred regenerated polyurethane foam ranges from 26 to 33 Kg·m^−3^, which is in line with the range of low density polyurethane foam. Furthermore, the range of compression strength was 100–320 KPa, the highest thermal conductivity was 0.02215 W·(m·K)^−1^, and the lowest was 0.01823 W·(m·K)^−1^.

Meanwhile, to further investigate the effects of the single- and two-component alcohololytic agents of propylene glycol and ethylene glycol on the degradation materials, propylene glycol and ethylene glycol were compounded to form a two-component alcohololytic agent. Moreover, the single-component and two-component control groups were designed for the comparison experiments. The reaction conditions were as follows: two groups of single-component propylene glycol and ethylene glycol compounding, as well as propylene glycol and ethylene glycol compounding were selected as the investigating factors for the alcohololytic agent. The mass ratio of propylene glycol and ethylene glycol compounding to degrade the waste polyurethane foam and alcohololytic agent was 1:1, the mass ratio of ethylene glycol to propylene glycol in the two-component alcohololytic agent was 6:4, ethylene glycol 60 g, propylene glycol 40 g, and the mass of ethylene glycol and propylene glycol in the single component was 100 g. The catalyst NaOH was added at 0.5%, the reaction temperature was 160 °C, and the reaction time was 3 h. The molecular weight of the degradation material was tested by gel permeation chromatography, and the viscosity and hydroxyl value of the degradation material were also tested.

**Figure 5:**
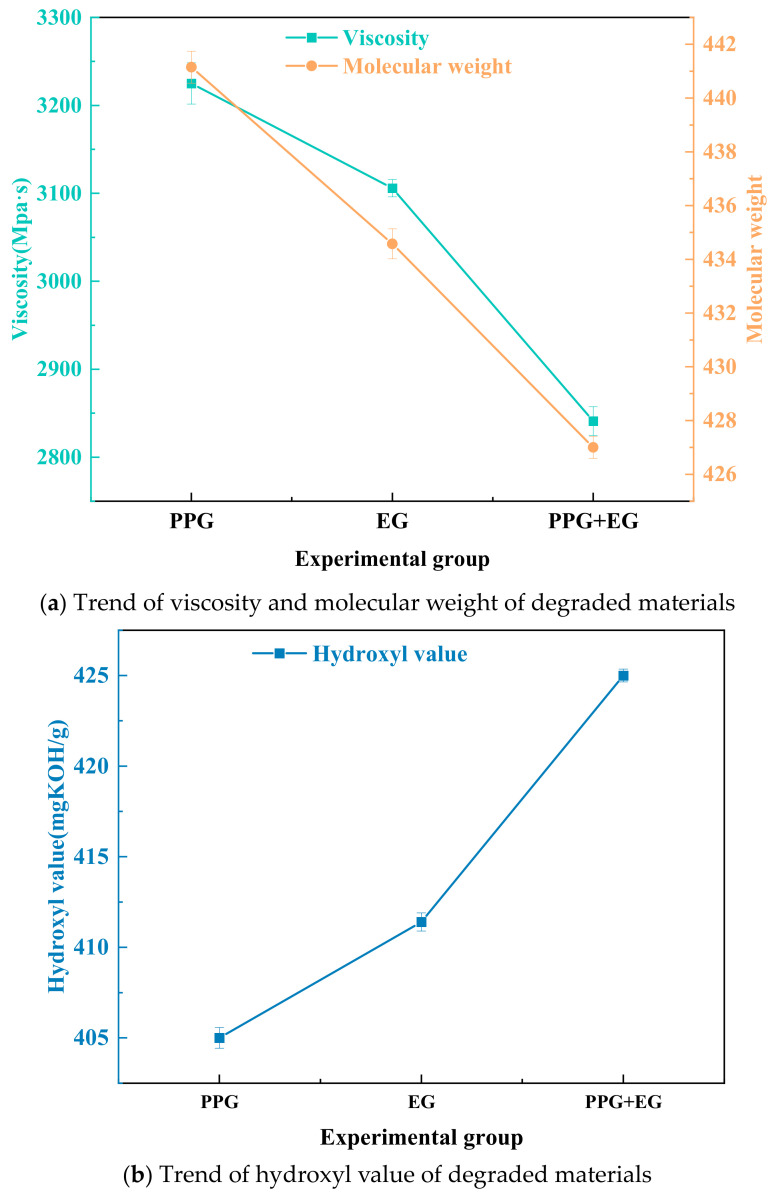
The effect of alcoholic solvents on the performance of degraded materials.

In Figure 5, the molecular weight, viscosity, and hydroxyl value of the degraded materials with different alcoholic solvents are given to identify the impact of alcoholic solvents on the degradation of waste polyurethane. It can be observed that the viscosity of the degraded material obtained after the alcoholysis of waste polyurethane shows a positive correlation with the molecular weight, and that the viscosity shows a negative correlation with the hydroxyl value. The lower the viscosity of the degraded material, the lower the molecular weight. The reason for this is that the more thoroughly the waste polyurethane is degraded, the more polyether polyol is obtained, and therefore the hydroxyl value will be higher. From the trend of the curves shown in Figure 5, it can be seen that the degradation material—in terms of viscosity, molecular weight, and the hydroxyl value of the degradation material—prepared via an alcoholysis using two-component alcohololytic agents of propylene glycol and ethylene glycol is better than the degradation material prepared by the one-component alcohololytic agents of propylene glycol and ethylene glycol. The above results illustrate that the alcoholysis of waste polyurethane by two-component alcoholysis agents is more complete. This is because, on the one hand, when propylene glycol and ethylene glycol are compounded as alcoholysis agents, the reactivity of propylene glycol can be reduced by reducing the content of propylene glycol in the matrix to reduce the proceeding side reaction. On the other hand, the high activity in ethylene glycol promotes the proceeding degradation reaction and increases the end-group hydroxyl value.

Therefore, this paper adopts the A2 group ethylene glycol and propylene glycol compound, where the best ratio was found to be 3:2. At the time, the degradation was rapid and the reaction was complete. Furthermore, at the same time, there was no excessive degradation. The size and strength of the foam when re-foaming were acceptable, which meant it was good for practical application in industrial production.

### 3.4. Effect of Catalysts on the Degradation of Waste Polyurethane

The catalysts play a crucial role in the degradation process of waste polyurethane. Efficient catalysts can greatly influence the whole reaction process without affecting the final product, reduce the reaction time of alcoholysis of waste polyurethane, and then improve its degradation efficiency, which provides a tremendous boost to the industrial promotion of waste polyurethane recycling.

The above study showed that the degradation effect was better when ethylene glycol was compounded with propylene glycol at a ratio of 3:2. In order to maximize the difference of degradation products after adding different catalysts, the alcohol solubilizer that was used was ethylene glycol. Propylene glycol was used at a ratio of 3:2, and the change in viscosity of the degraded material, as well as the change in compression strength and density of the produced foam, were investigated using different catalyst environments. In this experiment, a home-made bimetallic high-efficiency catalyst DMC was used, and the catalytic activity of the DMC catalyst was analyzed for application. The hydroxyl value of the degraded material was used as an evaluation index to study the effects of the traditional NaOH catalyst. In addition, the DMC catalyst and its two-component catalyst on the degradation process of waste polyurethane foam were also studied. The reaction conditions of the experiment were as follows: First, set up three experimental groups, then the catalysts are selected with the addition of NaOH at 0.5%, the addition of NaOH and DMC at 0.25% and 0.04%, respectively, as well as the addition of DMC at 0.04%. The mass ratio of waste polyurethane foam to the two-component alcoholic solvents was 1:1, the reaction temperature was 160 °C, and the reaction time was 2 h. Since the catalyst was used to investigate the catalytic rate of the polyurethane degradation process, the hydroxyl value was measured in order to analyze the reaction, which was not completely carried out. The pre-experiment showed that the complete reaction time was approximately 3 h—as such, the reaction time was set at 2 h.

**Figure 6:**
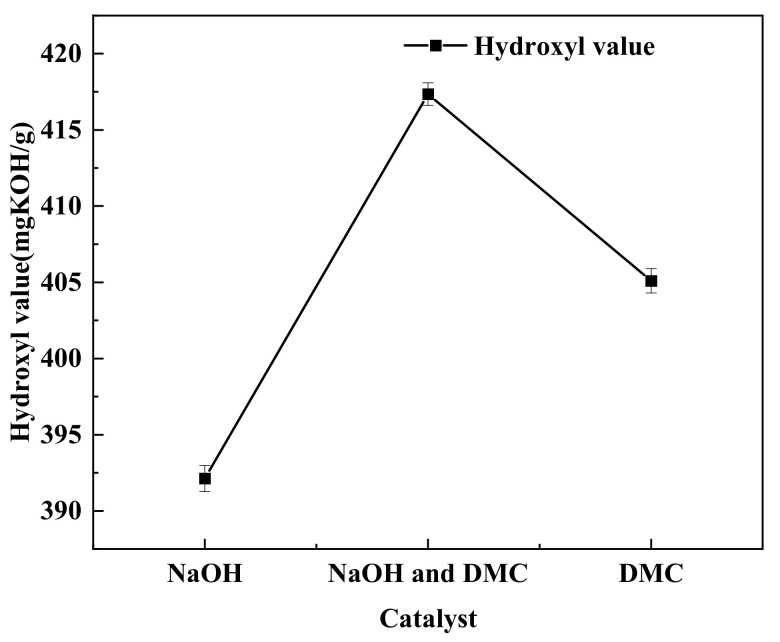
The effect of catalyst on hydroxyl value of degraded material.

The experimental results showed that the degradation materials all appeared brownish. Furthermore, the prepared foam samples were smooth in shape and white in color, and had high static compression strength. The foam samples turned out to be tight and not loose. In Figure 6, the variation in the hydroxyl values of the degraded material, after the catalytic degradation of waste polyurethane under the action of different catalysts, is given. The results revealed that the hydroxyl value of the degraded material, which was obtained from the alcoholysis reaction of waste polyurethane via the conventional NaOH catalyst, was less than that of the degraded material from the DMC catalyst. This explains the higher catalytic activity and the faster catalytic rate of the DMC catalyst for waste polyurethane foam, and why the waste polyurethane was degraded completely in the same amount of time. However, the hydroxyl value of the degraded material after the degradation of waste polyurethane with the DMC catalyst was smaller than that of the degraded material after the degradation with a two-component catalyst was blended with DMC and NaOH. The major reason for this was that, although the catalytic activity of the DMC catalyst was strong, the amount of the DMC catalyst added in the reaction system was small and thus the number of bases in the reaction system was also small. Moreover, the waste polyurethane foam at the beginning of the reaction was solid, and the reaction contact area between the DMC catalyst and the waste polyurethane foam was small due to the small number of bases, which leads to a greater decrease in the catalytic performance. Therefore, the mixing of a small amount of NaOH will play a transitional role in this stage, helping the DMC catalyst to degrade the waste polyurethane foam into a more liquid solid–liquid mixture, thus increasing the contact area between the DMC catalyst and the degraded material. The DMC catalyst will then play the original efficient catalytic role and greatly improve the degradation rate. Therefore, NaOH and DMC have the best effect of catalytic degradation when they are compounded.

**Figure 7:**
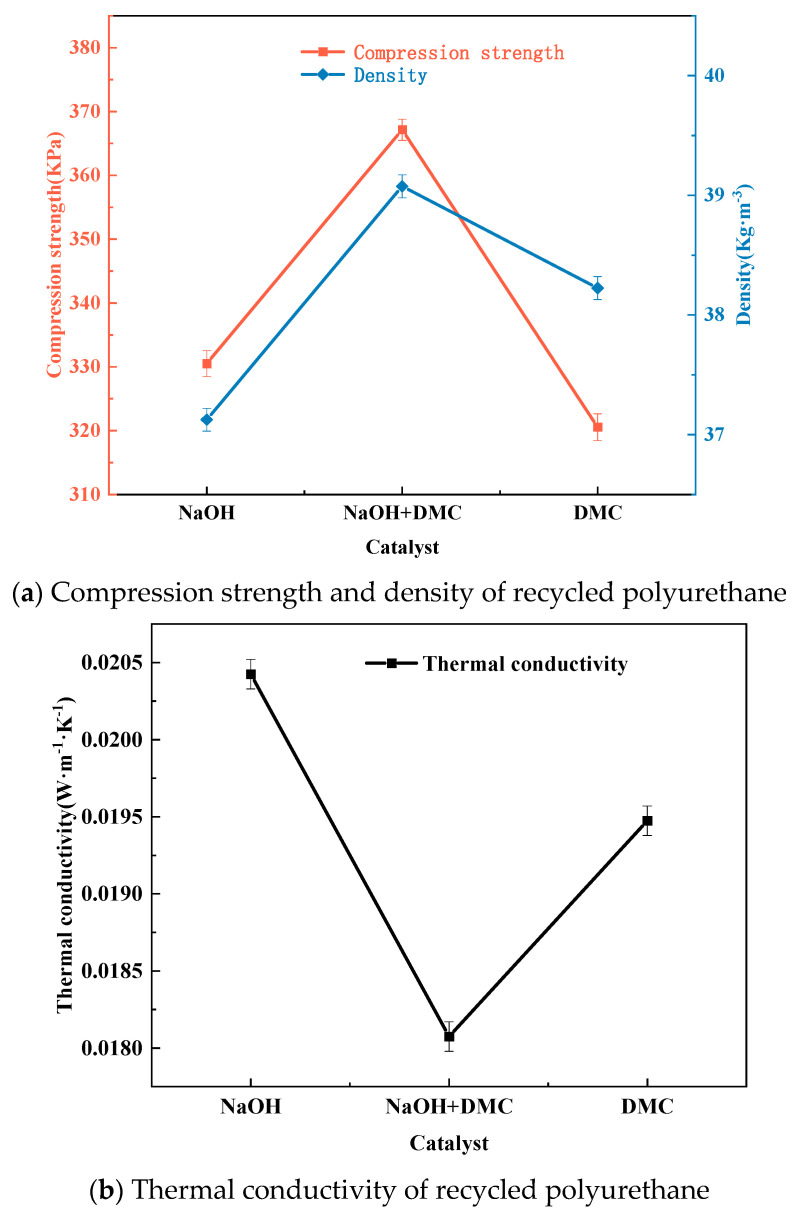
The effect of catalyst on performance of recycled polyurethane.

The different degraded materials were foamed, and the regenerated polyurethane foams were tested for their thermophysical properties to determine if there was a catalyst effect on the performance of the regenerated polyurethane. The trends of density, compression strength, and the thermal conductivity of the recycled polyurethane foam prepared from different catalyst degradation products are shown in Figure 7. The trends of density, compression strength, and thermal conductivity of the regenerated polyurethane foams prepared from different catalyst degradation products are shown in Figure 7. From the Figure 7, it can be seen that, with the change in the catalyst, the change in the trends of density and compression strength of the regenerated polyurethane foam are similar. Furthermore, both of them are the ones with the highest density and the strongest compression strength of the sample, which were obtained after the synergistic catalytic degradation and re-foaming of DMC and NaOH. However, the change in trends regarding thermal conductivity were found to be the opposite, i.e., they had the lowest thermal conductivity of the sample, which was obtained after the synergistic catalytic degradation and re-foaming of DMC and NaOH. The apparent density of the preferred regenerated polyurethane foam ranged from 35 to 40 Kg·m^−3^, which was in line with the range of low-density polyurethane foam; the range of the compression strength was 0.32–0.37 MPa, the highest thermal conductivity was 0.0205 W·m^−1^·K^−1^, and the lowest was 0.0180 W·m^−1^·K^−1^.

**Figure 8:**
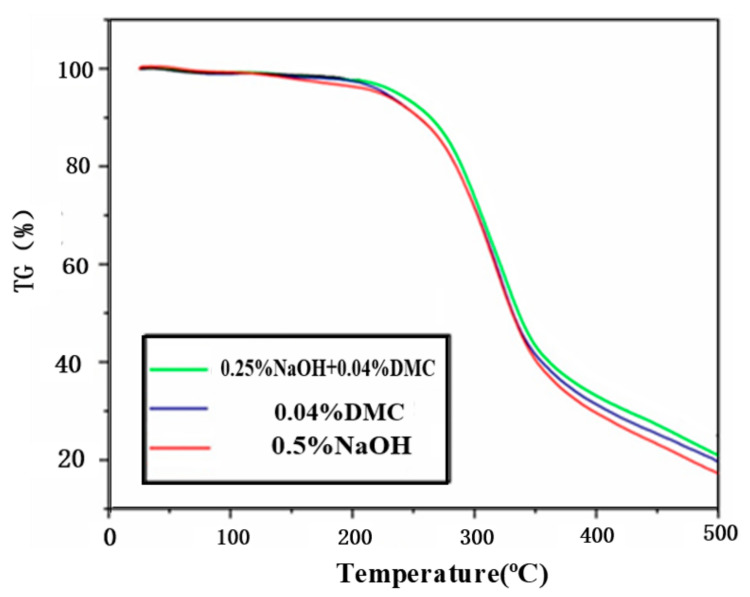
TG curves of regenerated polyurethane under different catalyst degradation systems.

To further study the thermal stability of the polymer, thermogravimetry was performed on the regenerated polyurethane after the degradation of the waste polyurethane was re-foamed by the three catalytic degradation systems, consisting of NaOH, DMC, and the catalysts compounded with both. In addition, the TG curves of the foamed materials were degraded by different catalysts for waste polyurethane, as is shown in Figure 8. The TG curves in the Figure 8 show the thermal weight loss rates of the regenerated polyurethane at different degradation temperatures, which were used to study the thermal stability of the regenerated polyurethane.

From Figure 8, the following conclusions can be drawn: the heat loss curve of polyurethane regenerated after the degradation of waste polyurethane mainly consists of two stages of heat loss processes, 161~290 °C and 325~418 °C. This is mainly because polyurethane has two types of structures, soft and hard segments. The mass loss in the first stage mainly comes from the decomposition of moisture and hard chain segments in the regenerated polyurethane. The mass loss in the second stage is mainly due to the decomposition of the soft segments in the regenerated polyurethane.

From the stage of 161 to 290 °C, it can be found that the initial decomposition temperature of the regenerated polyurethane, which was prepared with an addition of 0.5% NaOH, is 155 °C. Meanwhile, the initial decomposition temperature of the regenerated polyurethane, which was prepared with an addition of DMC 0.04%, is 217 °C, indicating that the thermal stability of the regenerated polyurethane prepared by the DMC system will be improved. From the second stage (326~415 °C), it can be concluded that the thermal weight loss temperature of the regenerated polyurethane prepared by the DMC system was significantly increased when compared to the NaOH system. In contrast, in the NaOH and DMC synergistic degradation system, the thermal weight loss temperature of the prepared regenerated polyurethane was increased more evidently, and the rate of the thermal degradation was also decreased significantly, indicating that the thermal stability of the regenerated polyurethane was improved. The reasons for this are mainly due to two aspects: on the one hand, the larger hydroxyl value in the regenerated polyether polyol prepared by DMC system makes the hard segment part of the prepared regenerated polyurethane larger, which further improves the spatial crosslinking density in the regenerated polyurethane; on the other hand, the thermal weight loss rate of the regenerated polyurethane increases with the increase in the addition of NaOH, as, although the increase in NaOH catalyst is small, it will work with the DMC catalyst synergistically in order to improve the thermal stability of the regenerated polyurethane. Therefore, the synergistic catalytic system of DMC and NaOH can enhance the thermal stability performance of regenerated polyurethane to a certain extent.

**Figure 9:**
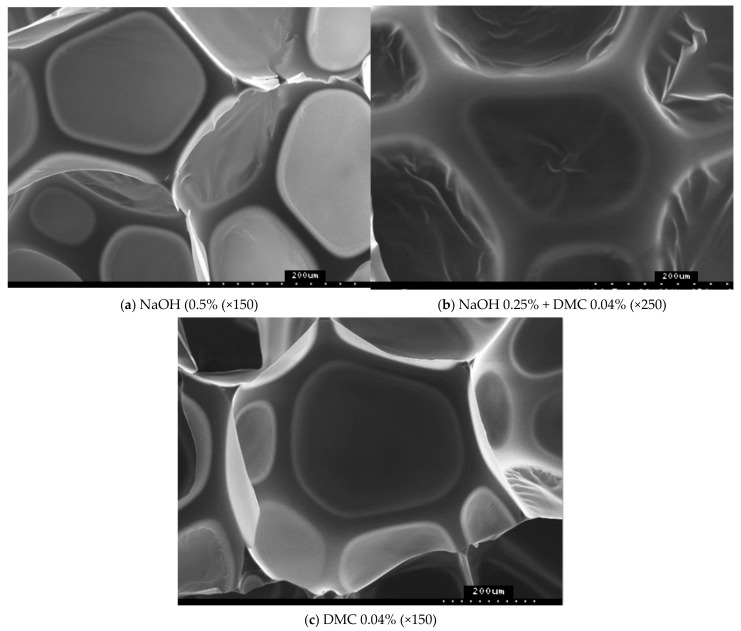
The SEM of regenerated polyurethane with different catalyst systems.

To observe the effect of different catalysts on the regenerated polyurethane more visually, the regenerated polyurethane samples that showed better comprehensive performance with the NaOH and DMC catalysts, as well as in both of their synergistic catalytic systems, were selected and cut into thin slices. Then, the complete area was selected for observation using a scanning electron microscope. The observation results are shown in Figure 9.

From the SEM Figure 9a–c above, it was found that in the NaOH system, the waste polyurethane produced by-products during the degradation process, which affected the performance of the regenerated polyurethane. This resulted in an incomplete blister structure of the regenerated polyurethane, a non-stout skeleton, and a relatively thin blister wall thickness. The molecular chain of waste polyurethane in the DMC system was not completely broken and the alcoholysis reaction was not complete, which also lead to the incomplete bubble structure of the prepared waste polyurethane. However, when the DMC and NaOH synergistic catalytic system was used, the bubble pores of the regenerated polyurethane prepared by both synergistic catalyses were relatively intact and regular. In addition, the bubble pore size was relatively uniform throughout the field of view, indicating that the foam was relatively stable during the foaming process. Moreover, the pore walls of the regenerated polyurethane prepared by the synergistic catalysis of DMC and NaOH were thicker and more uniform, and the geometry of the skeleton was better, indicating that the regenerated polyurethane foam had good cross-linkage by which to provide a good compressive strength to the foam. The mechanical properties of the regenerated polyurethane were the best with this catalytic system. Additionally, in the field of view, the foam integrity was found to be very high, the film formation was better, and the degree of closed pores in the foam was relatively high. The closed porosity had a great influence on the thermal insulation properties of the regenerated polyurethane foam. Therefore, the DMC and NaOH synergistic catalysis could prepare the regenerated polyurethane foams with excellent properties.

### 3.5. Effect of Reaction Temperature on the Degradation of Waste Polyurethane

According to the above experimental results, two-component alcoholic solvents compounded with propylene glycol and ethylene glycol, as well as a catalyst for the synergistic catalytic degradation of waste polyurethane foam by NaOH and DMC were identified. Based on this, this subsection further explores the optimal environment for the degradation of waste polyurethane when using the reaction temperature as a variable in the degradation process. The reaction conditions of the experiments were as follows: five groups of reaction temperatures, in increments of 10 °C, of 140 °C, 150 °C, 160 °C, 170 °C, and 180 °C, were selected as the investigating factors. The mass ratio of waste polyurethane foam to two-component alcoholic solvents was 1:1, the addition of NaOH catalyst was 0.25%, the addition of DMC catalyst was 0.04%, and the reaction time was 3 h.

**Figure 10:**
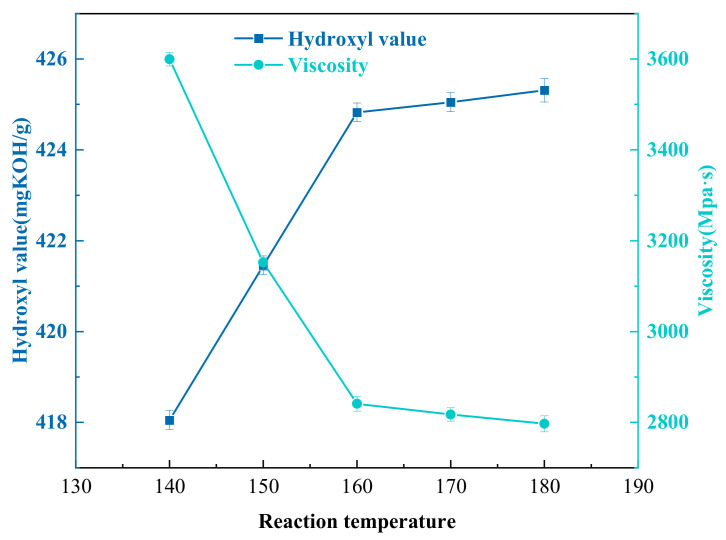
The effect of reaction temperature on hydroxyl value and viscosity of degraded material.

The effect of the reaction temperature on the degradation products during the degradation of waste polyurethane was analyzed by the viscosity and hydroxyl value of the degradation material (Figure 10). As seen in the Figure 10, as the reaction temperature increases, the viscosity of the degradation material decreases and the hydroxyl value increases, indicating that the degradation reaction is more than adequate, and that the waste polyurethane is degraded completely. However, it was found that there is a critical point with respect to the reaction temperature, which was found to be 160 °C. When the reaction temperature is 160 °C or higher, the changes in viscosity and hydroxyl value are not obvious, such that it can be concluded that the waste polyurethane foam is mainly alcoholized to polyether polyol at 160 °C. When the alcoholysis temperature is higher, the changes in molecular weight, viscosity, and hydroxyl value are not obvious, and the energy consumption also increases significantly; as such, 160 °C is the best alcoholysis temperature for waste polyurethane.

### 3.6. Effect of Reaction Time on the Degradation of Waste Polyurethane

After determining the reaction temperature of waste polyurethane alcoholysis, the effect of the alcoholysis reaction time as a variable on the degradation material of waste polyurethane foam was investigated. The reaction conditions for the experiments in this subsection were as follows: the reaction times were chosen as 1.5 h, 2 h, 2.5 h, 3 h, and 3.5 h as the influencing factors, the mass ratio of the waste polyurethane foam to the two-component alcoholysis agent was 1:1, the addition of NaOH was 0.25%, the addition of DMC was 0.04%, and the reaction temperature was 160 °C.

**Figure 11:**
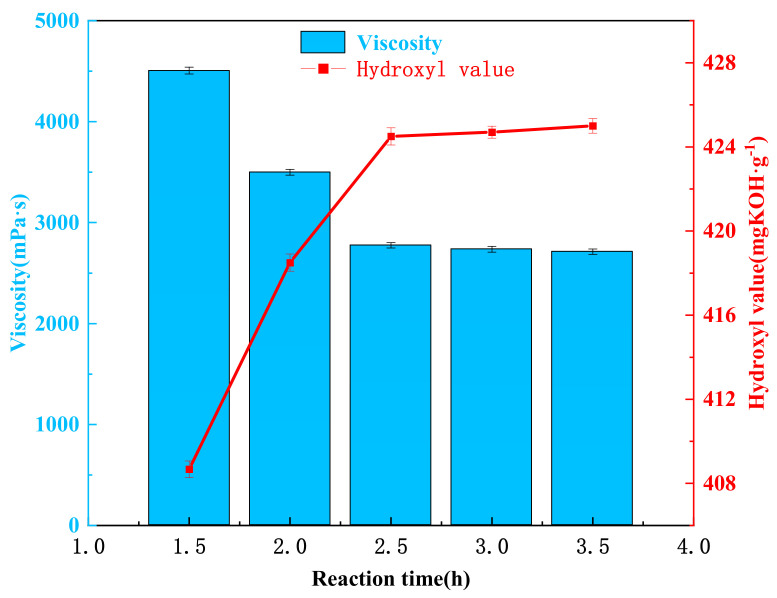
The viscosity and hydroxyl value of degradation material of waste polyurethane with different reaction time.

Figure 11 shows the quantitative comparison of the viscosity and hydroxyl values of degradation material of waste polyurethane with different reaction times. It can be found that the viscosity and hydroxyl values of the waste polyurethane degradation showed the opposite trend, with an increase in reaction time, with the viscosity of the waste polyurethane degradation gradually decreasing, and the hydroxyl value gradually increasing; however, the increase and decrease in these values gradually decreased. When the reaction reached 2.5 h, the decrease and increase in the viscosity and hydroxyl value of the waste polyurethane degradation material were negligible, indicating that the alcoholysis of the waste polyurethane foam was basically complete. From the viewpoint of production efficiency and cost, 2.5 h should be selected as the best alcoholysis reaction time.

### 3.7. Effect of Mass Ratio of Waste Polyurethane Foam to Two-Component Alcoholic Solvents on the Degradation of Waste Polyurethane

Finally, the effect of the mass ratio of waste polyurethane foam to the two-component alcoholic solvents solubilizer on the degradation process, with all the other reaction conditions determined, of waste polyurethane foam was investigated. The experimental reaction conditions in this subsection are as follows: the mass ratios of the waste polyurethane foam and two-component alcoholic solvents were chosen as 1:0.5, 1:1, 1:1.5, and 1:2, respectively, the addition of NaOH was 0.25%, the DMC was 0.04%, the reaction temperature was 160 °C, and the reaction time was 2.5 h.

**Figure 12:**
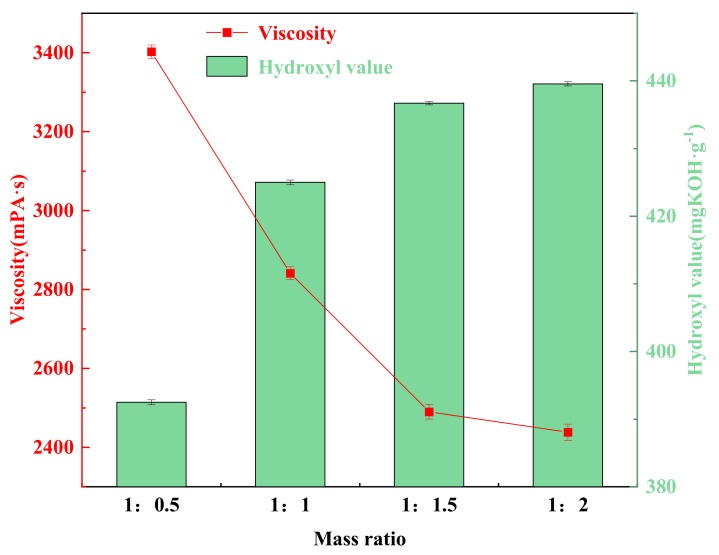
The viscosity and hydroxyl value of degradation material of waste polyurethane for different mass ratio.

For quantitatively analyzing the influence of the mass ratio, the viscosity and hydroxyl value of the degradation material of waste polyurethane was plotted (Figure 12) for the different mass ratios. As shown in Figure 12, the degradation completion rate (the degradation completion rate that is related to the viscosity and hydroxyl value of the degradation material, i.e., the higher the hydroxyl value and the lower the viscosity of the degradation material, then the higher the degradation completion rate) is proportional to the mass ratio of the waste polyurethane foam and the two-component alcoholic solvent. However, if the mass ratio is less than 1:1.5, then the difference in the degradation completion rate is not significant, and the raw material will be wasted. Therefore, the ratio of 1:1.5 was used.

### 3.8. Infrared Analysis of Waste Polyurethane Foam Degradation Material under Optimized Conditions

The main purpose of the degradation of the waste polyurethane foam was to recover polyol and to use it for the preparation of recycled polyurethane foam. This was performed in order to investigate whether the prepared degradation material was polyol or not. As such, infrared characterization tests of the degradation material were required. According to the previous paper, the optimal conditions for the various variables in the reaction were optimized, and the optimal scheme for the degradation of waste polyurethane was derived as follows: the mass ratio of the waste polyurethane foam to the two-component alcoholic solubilizer was 1:1.5, the addition of NaOH was 0.25%, the addition of DMC was 0.04%, the reaction temperature was 160 °C, and the reaction time was 2.5 h.

**Figure 13:**
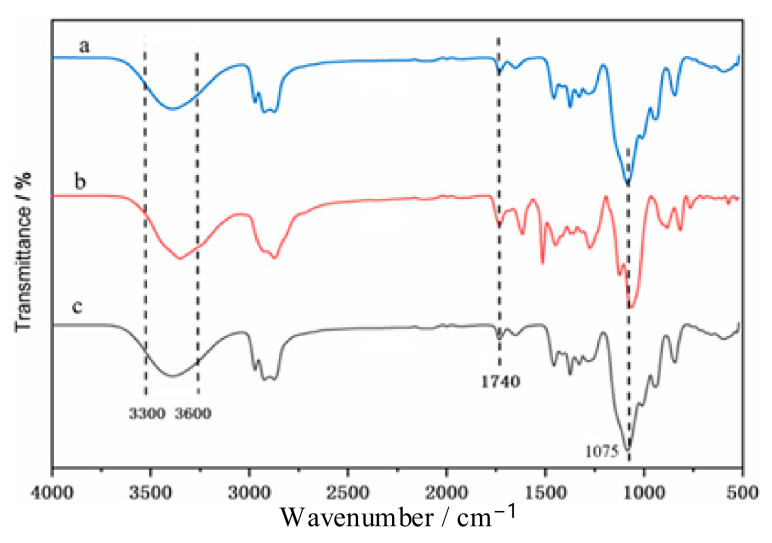
Infrared spectral curve of degraded material (a. 0.5% NaOH; b. 0.25% NaOH + 0.04% DMC; c. 0.04% DMC).

In Figure 13, there is a strong characteristic peak near 3200–3600 cm^−1^, which is the stretching vibration peak of the alcohol hydroxyl group. There is a strong characteristic peak near 1740 cm^−1^, which is the pan-frequency peak of benzene. There is also a clear characteristic peak near 1075 cm^−1^, which is the absorption band of the polyether polyurethane ether group. Therefore, it can be proved that the composition of the prepared degradation material is a mixture product of aromatic polyol and polyether polyol. Due to the presence of a rigid structural benzene ring in the aromatic polyol, the mechanical properties will be better than those of polyurethane foam made from pure polyether polyol in the preparation of recycled polyurethane foam. Therefore, this shows that the degradation material prepared by alcoholysis has a better performance than pure polyether polyol in foaming applications.

## 4. Conclusions

This paper investigated the degradation reaction of used polyurethane foam. The waste polyurethane foam was degraded to regenerated polyol, and regenerated polyurethane was prepared from the regenerated polyol. The scheme of the recycling and reuse of waste polyurethane was optimized, and the effects of different alcoholic solvents, catalysts, reaction temperatures, reaction times, and mass ratios between the waste polyurethane and alcoholic solvents on the performance of the proposed model were discussed. This was achieved by using the state of the degraded material, molecular weight, viscosity, hydroxyl value, and macroscopic morphology of the recycled polyurethane as evaluation indexes. Based on the results of the conducted studies, the following conclusions can be drawn.

In the process of the alcoholysis reaction of waste polyurethane, when two-component alcoholysis agents were used for the alcoholysis of waste polyurethane foam, the degradation material obtained had a good fluidity and higher hydroxyl value, which was better than the degradation material alcoholized by a one-component alcoholysis agent. Similarly, the NaOH and DMC synergistic catalytic system was the best for the degradation of waste polyurethane with a high system activity. Therefore, the best degradation scheme for waste polyurethane was as follows: 0.25% NaOH addition, 0.04% DMC addition, reaction temperature of 160 °C, and reaction time of 2.5 h. With these criteria, the alcoholysis of waste polyurethane foam was complete, and the prepared regenerated polyurethane had thick and uniform foam pore walls, a stout skeleton configuration, a high compressive strength, and good thermal stability.

## Figures and Tables

**Table 1:** The Proportion of EG and PPG.

Group Name	A1	A2	A3	A4
Proportion of EG and PPG	70:30	60:40	50:50	40:60

**Table 2:** Alcoholysis of used polyurethane foam degradation material situation.

Alcoholic Solvents	Color	Status	Mobility
Ethylene glycol	Sepia	Silty	Rather poor
Propylene glycol	Sepia	Silty	General

**Table 3:** Viscosity of degraded material and performance of recycled polyurethane foam of waste polyurethane under different ratios of alcoholic solvents.

Group Name	Mass Ratio of EG and PPG	Reaction Temperature/°C	Viscosity/Mpa·s	Compression Strength/Kpa	Molecular Weight	Density/kg·m^−3^	Thermal Conductivity/W·m^−1^·K^−1^
A1	7:3	160	3909.6	286.05	469	30.36	0.0202
A2	6:4	160	2842.2	317.2	427	32.4	0.01823
A3	5:5	160	1655.2	108.2	392	28.1	0.0215
A4	4:6	160	2984.1	104.4	431	26.075	0.02215

## Data Availability

The data presented in this study are available on request from the corresponding author. The data are not publicly available due to these data relate to the follow-up study.
